# *N*-(4-Chloro­benzo­yl)benzene­sulfonamide

**DOI:** 10.1107/S1600536809048399

**Published:** 2009-11-21

**Authors:** P. A. Suchetan, B. Thimme Gowda, Sabine Foro, Hartmut Fuess

**Affiliations:** a Department of Chemistry, Mangalore University, Mangalagangotri 574 199, Mangalore, India; b Institute of Materials Science, Darmstadt University of Technology, Petersenstrasse 23, D-64287 Darmstadt, Germany

## Abstract

In the crystal structure of the title compound, C_13_H_10_ClNO_3_S, the conformation of the N—H bond in the C—SO_2_—NH—C(O) segment is *anti* to the C=O bond. The dihedral angle between the two aromatic rings is 68.6 (1)°. The mol­ecule is twisted at the S atom with a dihedral angle of 75.7 (1)° between the sulfonyl benzene ring and the —SO_2_—NH—C—O segment; the dihedral angle between the latter and the benzoyl ring is 8.3 (2)°. In the crystal, mol­ecules are linked by N—H⋯O(S) hydrogen bonds.

## Related literature

For background literature and similar structures, see: Gowda *et al.* (2008 ▶, 2009**a* ▶,b* ▶).
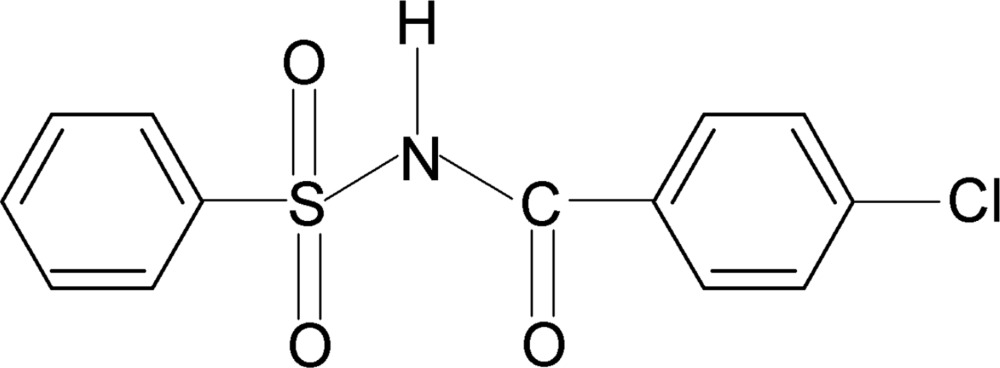


## Experimental

### 

#### Crystal data

C_13_H_10_ClNO_3_S
*M* *_r_* = 295.73
Triclinic, 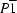

*a* = 5.4176 (4) Å
*b* = 10.717 (1) Å
*c* = 10.980 (1) Å
α = 86.666 (9)°
β = 83.903 (9)°
γ = 81.823 (8)°
*V* = 626.85 (9) Å^3^
*Z* = 2
Cu *K*α radiation
μ = 4.30 mm^−1^
*T* = 296 K
0.48 × 0.42 × 0.23 mm

#### Data collection

Enraf–Nonius CAD-4 diffractometer
Absorption correction: ψ scan (North *et al.*, 1968 ▶) *T*_min_ = 0.232, *T*_max_ = 0.438
2490 measured reflections
2233 independent reflections
2058 reflections with *I* > 2σ(*I*)
*R*_int_ = 0.014
3 standard reflections frequency: 120 min intensity decay: 1.5%

#### Refinement

*R*[*F* ^2^ > 2σ(*F* ^2^)] = 0.051
*wR*(*F* ^2^) = 0.152
*S* = 1.07
2233 reflections
173 parameters
H-atom parameters constrained
Δρ_max_ = 0.57 e Å^−3^
Δρ_min_ = −0.52 e Å^−3^

### 

Data collection: *CAD-4-PC* (Enraf–Nonius, 1996 ▶); cell refinement: *CAD-4-PC*; data reduction: *REDU4* (Stoe & Cie, 1987 ▶); program(s) used to solve structure: *SHELXS97* (Sheldrick, 2008 ▶); program(s) used to refine structure: *SHELXL97* (Sheldrick, 2008 ▶); molecular graphics: *PLATON* (Spek, 2009 ▶); software used to prepare material for publication: *SHELXL97*.

## Supplementary Material

Crystal structure: contains datablocks I, global. DOI: 10.1107/S1600536809048399/ng2687sup1.cif

Structure factors: contains datablocks I. DOI: 10.1107/S1600536809048399/ng2687Isup2.hkl

Additional supplementary materials:  crystallographic information; 3D view; checkCIF report

## Figures and Tables

**Table 1:** Hydrogen-bond geometry (Å, °)

*D*—H⋯*A*	*D*—H	H⋯*A*	*D*⋯*A*	*D*—H⋯*A*
N1—H1*N*⋯O1^i^	0.86	2.47	3.281 (3)	158

Symmetry code: (i) 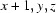.

**Fig. 1.**
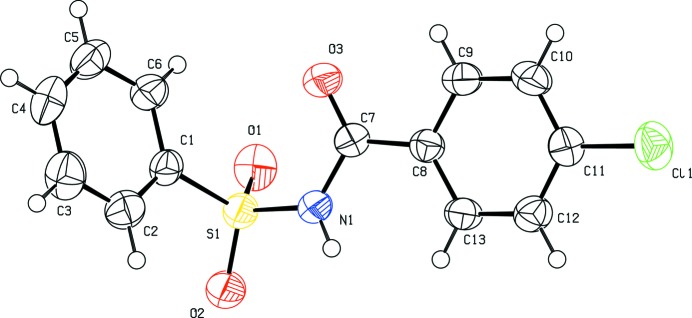
Molecular structure of (I), showing the atom labelling scheme and displacement ellipsoids are drawn at the 50% probability level.

**Fig. 2.**
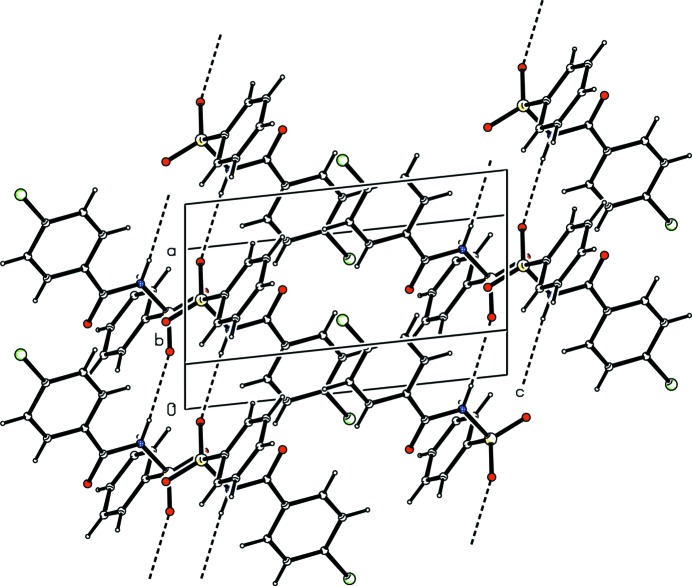
Molecular packing of (I) with hydrogen bonding shown as dashed lines.

## supplementary crystallographic information

### Comment

Diaryl acylsulfonamides are known as potent antitumor agents against a broad spectrum of human tumor xenografts (colon, lung, breast, ovary and prostate) in nude mice. As part of a study of the effect of ring and the side chain substituents on the crystal structures of *N*-aromatic sulfonamides (Gowda *et al.*, 2008, 2009*a,b*), in the present work, the structure of *N*-(4-chlorobenzoyl)benzenesulfonamide (I) has been determined (Fig.1). The conformation of the N—H bond in the C—SO_2_—NH—C(O) segment of the structure is *anti* to the C=O bond, similar to that observed in *N*-(benzoyl)benzenesulfonamide (II) (Gowda *et al.*, 2009*a*) and *N*-(3-chlorobenzoyl)- benzenesulfonamide (III)(Gowda *et al.*, 2009*b*). The molecule is twisted at the *S* atom with a dihedral angle of 75.7 (1)° between the sulfonyl benzene ring and the C—SO_2_—NH—C—O segment, compared to the values of 86.5(0.1) in (II) and 89.9 (1)° in (III). Furthermore, the dihedral angle between the two benzene rings is 68.6 (1)° in (I) and 80.3(0.1) in (II) and 87.5 (1)° in (III). The packing of molecules linked by of N—H···O(S) hydrogen bonds (Table 1) is shown in Fig. 2.

### Experimental

The title compound was prepared by refluxing a mixture of 4-chlorobenzoic acid, benzene sulfonamide and phosphorous oxy chloride for 5 h on a water bath. The resultant mixture was cooled and poured into ice cold water. The Solid obtained was filtered, washed thoroughly with water and then dissolved in sodium bicarbonate solution. The compound was later reprecipitated by acidifying the filtered solution with dilute HCl. The filtered and dried solid was recrystallized to the constant melting point.

Rod like colourless single crystals of the title compound were obtained from a slow evaporation of its toluene solution at room temperature and the X-ray diffraction studies were also carried out at room temperature.

### Refinement

The H atoms were positioned with idealized geometry using a riding model [N—H = 0.86 Å, C—H = 0.93 Å] and were refined with isotropic displacement parameters (set to 1.2 times of the *U*_eq_ of the parent atom).

### Crystal data

**Table:**

C_13_H_10_ClNO_3_S	*Z* = 2
*M**_r_* = 295.73	*F*(000) = 304
Triclinic, *P*1	*D*_x_ = 1.567 Mg m^−^^3^
Hall symbol: -P 1	Cu *K*α radiation, λ = 1.54180 Å
*a* = 5.4176 (4) Å	Cell parameters from 25 reflections
*b* = 10.717 (1) Å	θ = 5.9–21.0°
*c* = 10.980 (1) Å	µ = 4.30 mm^−^^1^
α = 86.666 (9)°	*T* = 296 K
β = 83.903 (9)°	Rod, colourless
γ = 81.823 (8)°	0.48 × 0.42 × 0.23 mm
*V* = 626.85 (9) Å^3^	

### Data collection

**Table:**

Enraf–Nonius CAD-4 diffractometer	2058 reflections with *I* > 2σ(*I*)
Radiation source: fine-focus sealed tube	*R*_int_ = 0.014
graphite	θ_max_ = 66.9°, θ_min_ = 4.1°
ω/2θ scans	*h* = 0→6
Absorption correction: ψ scan (North *et al.*, 1968)	*k* = −12→12
*T*_min_ = 0.232, *T*_max_ = 0.438	*l* = −13→13
2490 measured reflections	3 standard reflections every 120 min
2233 independent reflections	intensity decay: 1.5%

### Refinement

**Table:**

Refinement on *F*^2^	Secondary atom site location: difference Fourier map
Least-squares matrix: full	Hydrogen site location: inferred from neighbouring sites
*R*[*F*^2^ > 2σ(*F*^2^)] = 0.051	H-atom parameters constrained
*wR*(*F*^2^) = 0.152	*w* = 1/[σ^2^(*F*_o_^2^) + (0.1034*P*)^2^ + 0.2756*P*] where *P* = (*F*_o_^2^ + 2*F*_c_^2^)/3
*S* = 1.07	(Δ/σ)_max_ = 0.001
2233 reflections	Δρ_max_ = 0.57 e Å^−^^3^
173 parameters	Δρ_min_ = −0.52 e Å^−^^3^
0 restraints	Extinction correction: *SHELXL97* (Sheldrick, 2008), Fc^*^=kFc[1+0.001xFc^2^λ^3^/sin(2θ)]^-1/4^
Primary atom site location: structure-invariant direct methods	Extinction coefficient: 0.063 (5)

### Special details

**Table:**

Geometry. All e.s.d.'s (except the e.s.d. in the dihedral angle between two l.s. planes) are estimated using the full covariance matrix. The cell e.s.d.'s are taken into account individually in the estimation of e.s.d.'s in distances, angles and torsion angles; correlations between e.s.d.'s in cell parameters are only used when they are defined by crystal symmetry. An approximate (isotropic) treatment of cell e.s.d.'s is used for estimating e.s.d.'s involving l.s. planes.
Refinement. Refinement of *F*^2^ against ALL reflections. The weighted *R*-factor *wR* and goodness of fit *S* are based on *F*^2^, conventional *R*-factors *R* are based on *F*, with *F* set to zero for negative *F*^2^. The threshold expression of *F*^2^ > σ(*F*^2^) is used only for calculating *R*-factors(gt) *etc*. and is not relevant to the choice of reflections for refinement. *R*-factors based on *F*^2^ are statistically about twice as large as those based on *F*, and *R*- factors based on ALL data will be even larger.

### Fractional atomic coordinates and isotropic or equivalent isotropic displacement parameters (Å^2^)

**Table:**

	*x*	*y*	*z*	*U*_iso_*/*U*_eq_	
C1	0.5435 (4)	0.1733 (2)	0.8771 (2)	0.0411 (6)	
C2	0.7400 (5)	0.0803 (3)	0.8980 (3)	0.0532 (7)	
H2	0.8601	0.0939	0.9485	0.064*	
C3	0.7552 (6)	−0.0338 (3)	0.8426 (3)	0.0630 (8)	
H3	0.8860	−0.0976	0.8562	0.076*	
C4	0.5779 (6)	−0.0531 (3)	0.7674 (3)	0.0583 (7)	
H4	0.5904	−0.1295	0.7296	0.070*	
C5	0.3821 (6)	0.0403 (3)	0.7480 (3)	0.0555 (7)	
H5	0.2622	0.0263	0.6976	0.067*	
C6	0.3620 (5)	0.1542 (3)	0.8027 (2)	0.0469 (6)	
H6	0.2292	0.2171	0.7900	0.056*	
C7	0.6462 (5)	0.4535 (2)	0.7460 (2)	0.0408 (6)	
C8	0.7940 (4)	0.5520 (2)	0.6881 (2)	0.0387 (5)	
C9	0.7425 (5)	0.5974 (3)	0.5720 (2)	0.0519 (7)	
H9	0.6201	0.5657	0.5343	0.062*	
C10	0.8682 (6)	0.6884 (3)	0.5111 (2)	0.0534 (7)	
H10	0.8306	0.7190	0.4332	0.064*	
C11	1.0517 (5)	0.7341 (2)	0.5673 (2)	0.0441 (6)	
C12	1.1054 (5)	0.6914 (3)	0.6829 (3)	0.0472 (6)	
H12	1.2279	0.7236	0.7201	0.057*	
C13	0.9770 (5)	0.6006 (2)	0.7438 (2)	0.0426 (6)	
H13	1.0125	0.5716	0.8224	0.051*	
N1	0.7013 (4)	0.4043 (2)	0.8612 (2)	0.0465 (5)	
H1N	0.8365	0.4200	0.8882	0.056*	
O1	0.2646 (4)	0.37670 (19)	0.95259 (19)	0.0590 (6)	
O2	0.6378 (5)	0.2966 (2)	1.05960 (18)	0.0655 (6)	
O3	0.4823 (4)	0.41570 (18)	0.69711 (17)	0.0524 (5)	
Cl1	1.21303 (15)	0.84797 (7)	0.48922 (7)	0.0649 (3)	
S1	0.51961 (12)	0.31704 (6)	0.94915 (5)	0.0456 (3)	

### Atomic displacement parameters (Å^2^)

**Table:**

	*U*^11^	*U*^22^	*U*^33^	*U*^12^	*U*^13^	*U*^23^
C1	0.0404 (12)	0.0423 (12)	0.0425 (13)	−0.0149 (10)	−0.0024 (10)	0.0009 (10)
C2	0.0439 (13)	0.0557 (15)	0.0621 (16)	−0.0112 (11)	−0.0109 (12)	−0.0003 (12)
C3	0.0506 (16)	0.0514 (16)	0.084 (2)	−0.0013 (13)	−0.0019 (15)	−0.0026 (15)
C4	0.0625 (17)	0.0472 (14)	0.0670 (19)	−0.0216 (13)	0.0094 (14)	−0.0117 (13)
C5	0.0557 (16)	0.0611 (17)	0.0556 (16)	−0.0258 (13)	−0.0056 (12)	−0.0081 (13)
C6	0.0426 (13)	0.0506 (14)	0.0499 (14)	−0.0117 (11)	−0.0082 (11)	−0.0012 (11)
C7	0.0434 (12)	0.0408 (12)	0.0395 (12)	−0.0072 (10)	−0.0069 (10)	−0.0038 (10)
C8	0.0381 (12)	0.0398 (12)	0.0383 (12)	−0.0042 (9)	−0.0063 (9)	−0.0011 (9)
C9	0.0573 (15)	0.0600 (16)	0.0433 (14)	−0.0180 (13)	−0.0168 (12)	0.0031 (12)
C10	0.0609 (16)	0.0624 (16)	0.0395 (13)	−0.0154 (13)	−0.0141 (11)	0.0104 (12)
C11	0.0405 (12)	0.0437 (13)	0.0462 (13)	−0.0046 (10)	0.0013 (10)	0.0000 (10)
C12	0.0408 (13)	0.0540 (14)	0.0487 (14)	−0.0125 (11)	−0.0068 (10)	−0.0003 (11)
C13	0.0410 (12)	0.0507 (14)	0.0373 (12)	−0.0086 (10)	−0.0094 (10)	0.0024 (10)
N1	0.0553 (12)	0.0473 (11)	0.0426 (12)	−0.0213 (10)	−0.0150 (9)	0.0043 (9)
O1	0.0572 (12)	0.0569 (11)	0.0598 (12)	−0.0047 (9)	0.0063 (9)	−0.0078 (9)
O2	0.0992 (17)	0.0649 (13)	0.0407 (11)	−0.0339 (12)	−0.0190 (10)	0.0066 (9)
O3	0.0527 (11)	0.0588 (11)	0.0511 (10)	−0.0204 (9)	−0.0174 (8)	0.0049 (8)
Cl1	0.0664 (5)	0.0603 (5)	0.0678 (5)	−0.0208 (3)	0.0014 (4)	0.0134 (4)
S1	0.0579 (5)	0.0457 (4)	0.0362 (4)	−0.0166 (3)	−0.0057 (3)	−0.0003 (3)

### Geometric parameters (Å, °)

**Table:**

C1—C2	1.379 (4)	C8—C9	1.381 (3)
C1—C6	1.387 (4)	C8—C13	1.393 (4)
C1—S1	1.755 (2)	C9—C10	1.371 (4)
C2—C3	1.385 (4)	C9—H9	0.9300
C2—H2	0.9300	C10—C11	1.383 (4)
C3—C4	1.376 (5)	C10—H10	0.9300
C3—H3	0.9300	C11—C12	1.371 (4)
C4—C5	1.376 (5)	C11—Cl1	1.735 (3)
C4—H4	0.9300	C12—C13	1.377 (4)
C5—C6	1.377 (4)	C12—H12	0.9300
C5—H5	0.9300	C13—H13	0.9300
C6—H6	0.9300	N1—S1	1.653 (2)
C7—O3	1.212 (3)	N1—H1N	0.8600
C7—N1	1.387 (3)	O1—S1	1.435 (2)
C7—C8	1.488 (3)	O2—S1	1.422 (2)
			
C2—C1—C6	121.3 (2)	C10—C9—C8	121.3 (2)
C2—C1—S1	119.1 (2)	C10—C9—H9	119.4
C6—C1—S1	119.5 (2)	C8—C9—H9	119.4
C1—C2—C3	118.8 (3)	C9—C10—C11	118.9 (2)
C1—C2—H2	120.6	C9—C10—H10	120.5
C3—C2—H2	120.6	C11—C10—H10	120.5
C4—C3—C2	120.3 (3)	C12—C11—C10	121.0 (2)
C4—C3—H3	119.8	C12—C11—Cl1	120.2 (2)
C2—C3—H3	119.8	C10—C11—Cl1	118.8 (2)
C3—C4—C5	120.1 (3)	C11—C12—C13	119.7 (2)
C3—C4—H4	119.9	C11—C12—H12	120.2
C5—C4—H4	119.9	C13—C12—H12	120.2
C4—C5—C6	120.6 (3)	C12—C13—C8	120.2 (2)
C4—C5—H5	119.7	C12—C13—H13	119.9
C6—C5—H5	119.7	C8—C13—H13	119.9
C5—C6—C1	118.8 (3)	C7—N1—S1	123.18 (18)
C5—C6—H6	120.6	C7—N1—H1N	118.4
C1—C6—H6	120.6	S1—N1—H1N	118.4
O3—C7—N1	119.6 (2)	O2—S1—O1	119.60 (14)
O3—C7—C8	122.9 (2)	O2—S1—N1	103.57 (12)
N1—C7—C8	117.5 (2)	O1—S1—N1	109.22 (12)
C9—C8—C13	118.9 (2)	O2—S1—C1	109.18 (12)
C9—C8—C7	116.9 (2)	O1—S1—C1	108.42 (12)
C13—C8—C7	124.2 (2)	N1—S1—C1	106.02 (11)
			
C6—C1—C2—C3	0.4 (4)	C10—C11—C12—C13	0.7 (4)
S1—C1—C2—C3	178.9 (2)	Cl1—C11—C12—C13	−179.7 (2)
C1—C2—C3—C4	0.4 (5)	C11—C12—C13—C8	0.2 (4)
C2—C3—C4—C5	−0.8 (5)	C9—C8—C13—C12	−0.8 (4)
C3—C4—C5—C6	0.5 (5)	C7—C8—C13—C12	179.8 (2)
C4—C5—C6—C1	0.3 (4)	O3—C7—N1—S1	−12.7 (3)
C2—C1—C6—C5	−0.8 (4)	C8—C7—N1—S1	167.24 (17)
S1—C1—C6—C5	−179.2 (2)	C7—N1—S1—O2	−175.7 (2)
O3—C7—C8—C9	−1.9 (4)	C7—N1—S1—O1	−47.2 (2)
N1—C7—C8—C9	178.1 (2)	C7—N1—S1—C1	69.4 (2)
O3—C7—C8—C13	177.6 (3)	C2—C1—S1—O2	−26.5 (3)
N1—C7—C8—C13	−2.4 (4)	C6—C1—S1—O2	151.9 (2)
C13—C8—C9—C10	0.3 (4)	C2—C1—S1—O1	−158.4 (2)
C7—C8—C9—C10	179.8 (3)	C6—C1—S1—O1	20.1 (2)
C8—C9—C10—C11	0.7 (4)	C2—C1—S1—N1	84.5 (2)
C9—C10—C11—C12	−1.2 (4)	C6—C1—S1—N1	−97.1 (2)
C9—C10—C11—Cl1	179.3 (2)		

### Hydrogen-bond geometry (Å, °)

**Table:**

*D*—H···*A*	*D*—H	H···*A*	*D*···*A*	*D*—H···*A*
N1—H1N···O1^i^	0.86	2.47	3.281 (3)	158

Symmetry codes: (i) *x*+1, *y*, *z*.
